# Correction: Natural cycle versus hormone replacement therapy as endometrial preparation in ovulatory women undergoing frozen-thawed embryo transfer: The COMPETE open-label randomized controlled trial

**DOI:** 10.1371/journal.pmed.1004856

**Published:** 2025-12-09

**Authors:** The PLOS Medicine Staff

## Notice of Republication

This article was republished on November 18, 2025, to correct an error in the title that was introduced during the typesetting process. The publisher apologizes for the error. Please download this article again to view the correct version. The originally published, uncorrected article and the republished, corrected articles are provided here for reference.

## Supporting information

S1 File Originally published, uncorrected article. (PDF)

S2 File Republished, corrected article. (PDF)
