# Supplementary material for: Natural cycle versus hormone replacement therapy as endometrial preparation in ovulatory women undergoing frozen-thawed embryo transfer: The COMPETE open-label randomized controlled trial
Source: PLoS Med. 2025 Jun 25;22(6):e1004630. doi: 10.1371/journal.pmed.1004630 (PMC12193059; doi:10.1371/journal.pmed.1004630)
Supplement: S1 Table — (DOCX) [file pmed.1004630.s001.docx]

S1 Table. Summary of Adverse Events

|  | **NC (n=448)** | **HRT (n=454)** | ***P*-value**^a^ |
| --- | --- | --- | --- |
| Adverse events | 15 (3.4) | 14 (3.1) | 0.822 |
| Nausea | 0 (0.0) | 2 (0.4) |  |
| Endometrial cavity fluid | 3 (0.7) | 2 (0.4) |  |
| Pelvic fluid | 5 (1.1) | 4 (0.9) |  |
| Upset stomach | 5 (1.1) | 0 (0.0) |  |
| Allergy | 0 (0.0) | 2 (0.4) |  |
| Dizziness | 0 (0.0) | 1 (0.2) |  |
| Vaginal bleeding | 2 (0.4) | 3 (0.7) |  |

NC, natural cycle; HRT, hormone replacement treatment.

^a^ Data are presented as n (%).
