# Supplementary material for: Natural cycle versus hormone replacement therapy as endometrial preparation in ovulatory women undergoing frozen-thawed embryo transfer: The COMPETE open-label randomized controlled trial
Source: PLoS Med. 2025 Jun 25;22(6):e1004630. doi: 10.1371/journal.pmed.1004630 (PMC12193059; doi:10.1371/journal.pmed.1004630)
Supplement: S2 Table — (DOCX) [file pmed.1004630.s002.docx]

S2 Table. Reproductive Outcomes (Per-Protocol Analysis)

| **Clinical outcomes** | **NC** | |  | **HRT** | | **Absolute difference/mean difference (95% CI)**^a^ | **Risk ratio (95% CI)**^a^ |
| --- | --- | --- | --- | --- | --- | --- | --- |
|  | **N** | **n(%)/mean(SD)** |  | **N** | **n(%)/mean(SD)** |  |  |
| Live birth | 343 | 198 (57.7) |  | 410 | 172 (42.0) | **15.8 (8.7, 22.9)** | **1.38 (1.19, 1.59)** |
| Endometrial thickness (mm) | 338 | 11.1 (1.6) |  | 405 | 10.3 (1.3) | **0.78 (0.56, 0.99)** | ― |
| Cycle cancellation | 343 | 8 (2.3) |  | 410 | 9 (2.2) | 0.1 (-2, 2.3) | 1.06 (0.41,2.72) |
| Biochemical pregnancy | 343 | 244 (71.1) |  | 410 | 246 (60.0) | **11.1 (4.4, 17.9)** | **1.19 (1.07, 1.32)** |
| Clinical pregnancy | 343 | 227 (66.2) |  | 410 | 234 (57.1) | **9.1 (2.2, 16.0)** | **1.16 (1.04, 1.30)** |
| Miscarriage | 227 | 24 (10.6) |  | 234 | 55 (23.5) | **-12.9 (-19.7, -6.2)** | **0.45 (0.29, 0.70)** |
| Ongoing pregnancy | 343 | 202 (59.0) |  | 410 | 178 (43.4) | **15.5 (8.4, 22.6)** | **1.36 (1.18, 1.56)** |
| Multiple pregnancy | 343 | 12 (3.5) |  | 410 | 15 (3.7) | -0.2 (-2.8, 2.5) | 0.96 (0.45, 2.02) |
| Ectopic pregnancy^*^ | 343 | 5 (1.5) |  | 410 | 7 (1.7) | -0.2 (-2, 1.5) | 0.85 (0.27, 2.67) |

NC, natural cycle; HRT, hormone replacement treatment; CI, confidence interval.

^a^ HRT group was regarded as the reference group.

^*^ Posthoc specified endpoints.
