# Supplementary material for: Natural cycle versus hormone replacement therapy as endometrial preparation in ovulatory women undergoing frozen-thawed embryo transfer: The COMPETE open-label randomized controlled trial
Source: PLoS Med. 2025 Jun 25;22(6):e1004630. doi: 10.1371/journal.pmed.1004630 (PMC12193059; doi:10.1371/journal.pmed.1004630)
Supplement: S3 Table — (DOCX) [file pmed.1004630.s003.docx]

S3 Table. Maternal and Perinatal Outcomes (Per-Protocol Analysis)

| **Clinical outcomes** | **NC** | |  | **HRT** | | **Absolute difference/mean difference (95% CI)**^a^ | **Risk ratio (95% CI)**^a^ |
| --- | --- | --- | --- | --- | --- | --- | --- |
|  | **N** | **n(%)/mean(SD)** |  | **N** | **n(%)/mean(SD)** |  |  |
| Maternal hyperthyroidism^*^ | 202 | 5 (2.5) |  | 178 | 4 (2.3) | 0.2 (-2.8, 3.3) | 1.10 (0.30, 4.04) |
| Maternal hypothyroidism^*^ | 202 | 25 (12.4) |  | 178 | 14 (7.9) | 4.5 (-1.5, 10.5) | 1.57 (0.84, 2.93) |
| Polyhydramnios^*^ | 197 | 12 (6.1) |  | 172 | 5 (2.9) | 3.2 (-1, 7.4) | 2.10 (0.75, 5.83) |
| Oligohydramnios^*^ | 197 | 3 (1.5) |  | 172 | 6 (3.5) | -2 (-5.2, 1.3) | 0.44 (0.11, 1.72) |
| Gestational diabetes mellitus | 202 | 22 (10.9) |  | 178 | 28(15.7) | -4.8 (-11.7, 2) | 0.69 (0.41, 1.17) |
| Hypertensive disorders of pregnancy | 202 | 16 (7.9) |  | 178 | 13 (7.3) | 0.6 (-4.7, 6) | 1.08 (0.54, 2.19) |
| Pregnancy-induced hypertension | 202 | 13 (6.4) |  | 178 | 10 (5.6) | 0.8 (-4, 5.6) | 1.15 (0.52, 2.55) |
| Pre-eclampsia | 202 | 3 (1.5) |  | 178 | 3 (1.7) | -0.2 (-2.7, 2.3) | 0.88 (0.18, 4.31) |
| Antepartum haemorrhage | 197 | 25 (12.7) |  | 172 | 36 (20.9) | **-8.2 (-15.9, -0.6)** | **0.61 (0.38, 0.97)** |
| Placenta previa | 197 | 2 (1.0) |  | 172 | 2 (1.2) | -0.1 (-2.3, 2) | 0.87 (0.12, 6.13) |
| Placenta accreta | 197 | 19 (9.6) |  | 172 | 23 (13.4) | -3.7 (-10.3, 2.8) | 0.72 (0.41, 1.28) |
| Unexplained | 197 | 4 (2.0) |  | 172 | 11 (6.4) | **-4.4 (-8.5, -0.2)** | **0.32 (0.10, 0.98)** |
| Postpartum anemia^*^ | 198 | 14 (7.1) |  | 172 | 11 (6.4) | 0.7 (-4.4, 5.8) | 1.11 (0.52, 2.37) |
| Preterm birth | 202 | 13 (6.4) |  | 178 | 20 (11.2) | -4.8 (-10.5, 0.9) | 0.57 (0.29, 1.12) |
| Spontaneous | 202 | 8 (4.0) |  | 178 | 13 (7.3) | -3.3 (-8, 1.3) | 0.54 (0.23, 1.28) |
| Medical reasons | 202 | 5 (2.5) |  | 178 | 7 (3.9) | -1.5 (-5, 2.1) | 0.63 (0.20, 1.95) |
| PPROM^*^ | 197 | 8 (4.1) |  | 172 | 11 (6.4) | -2.3 (-6.9, 2.2) | 0.63 (0.26, 1.54) |
| Mode of delivery, cesarean section^*^ | 198 | 139 (70.2) |  | 172 | 136 (79.1) | -8.9 (-17.7, -0.1) | 0.89 (0.79, 1.00) |
| Gestational age at birth (weeks) ^*^ | 198 | 38.9 (1.7) ^b^ |  | 172 | 38.8 (1.9) ^c^ | 0.16 (-0.2, 0.53) | ― |
| Singleton |  |  |  |  |  |  |  |
| Birth weight (g) | 191 | 3376.0 (535.0) |  | 161 | 3342.4 (533.4) | 33.69 (-78.73, 146.11) | ― |
| Low birth weight (<2500g) | 191 | 11 (5.8) |  | 161 | 12 (7.5) | -1.7 (-6.9, 3.5) | 0.77 (0.35, 1.70) |
| Very low birth weight (<1500g) | 191 | 0 (0.0) |  | 161 | 1 (0.6) | -0.6 (-1.8, 0.6) | ― |
| High birth weight (>4000g) | 191 | 17 (8.9) |  | 161 | 13 (8.1) | 0.8 (-5, 6.7) | 1.10 (0.55, 2.20) |
| Very high birth weight (>4500g) | 191 | 3 (1.6) |  | 161 | 1 (0.6) | 0.9 (-1.2, 3.1) | 2.53 (0.27, 24.08) |
| Large for gestational age | 191 | 34 (17.8) |  | 161 | 25 (15.5) | 2.3 (-5.5, 10.1) | 1.15 (0.72, 1.84) |
| Small for gestational age | 191 | 9 (4.7) |  | 161 | 6 (3.7) | 1 (-3.2, 5.2) | 1.26 (0.46, 3.48) |
| Congenital anomaly | 191 | 5 (2.6) |  | 161 | 5 (3.1) | -0.5 (-4, 3) | 0.84 (0.25, 2.86) |
| Twins^b^ |  |  |  |  |  |  |  |
| Birth weight (g) | 14 | 2472.1 (452.4) |  | 22 | 2401.8 (410.3) | 70.33 (-295.94, 436.59) | ― |
| Low birth weight (<2500g) | 14 | 6 (42.9) |  | 22 | 12 (54.6) | -11.7 (-53.6, 30.2) | 0.79 (0.32, 1.92) |
| Very low birth weight (<1500g) | 14 | 0 (0.0) |  | 22 | 0 (0.0) | ― | ― |
| High birth weight (>4000g) | 14 | 0 (0.0) |  | 22 | 0 (0.0) | ― | ― |
| Very high birth weight (>4500g) | 14 | 0 (0.0) |  | 22 | 0 (0.0) | ― | ― |
| Large for gestational age | 14 | 2 (14.3) |  | 22 | 0 (0.0) | ― | ― |
| Small for gestational age | 14 | 5 (35.7) |  | 22 | 2 (9.1) | 26.6 (-2.5, 55.8) | 3.93 (0.88, 17.44) |
| Congenital anomaly | 14 | 4 (28.6) |  | 22 | 4 (18.2) | 10.4 (-31.3, 52.1) | 1.57 (0.27, 9.18) |
| NICU admission^*^ | 197 | 23 (11.7) |  | 172 | 25 (14.5) | -2.9 (-9.8, 4.1) | 0.80 (0.47, 1.36) |
| Perinatal mortality | 0 | 0 (0.0) |  | 0 | 0 (0.0) | ― | ― |

NC, natural cycle; HRT, hormone replacement treatment; CI, confidence interval; PPROM, preterm premature rupture of membranes; NICU, neonatal intensive care unit.

^a^ HRT group was regarded as the reference group.

^b^ Generalized estimating equation for twins. Data represent the number of twin pairs.

^*^ Posthoc specified endpoints.
