# Supplementary material for: Natural cycle versus hormone replacement therapy as endometrial preparation in ovulatory women undergoing frozen-thawed embryo transfer: The COMPETE open-label randomized controlled trial
Source: PLoS Med. 2025 Jun 25;22(6):e1004630. doi: 10.1371/journal.pmed.1004630 (PMC12193059; doi:10.1371/journal.pmed.1004630)
Supplement: S4 Table — (DOCX) [file pmed.1004630.s004.docx]

S4 Table. Sensitivity Analysis of Reproductive Outcomes by only including First Frozen-thawed Embryo Cycles (Intention-To-Treat)

| **Clinical outcomes** | **NC** | |  | **HRT** | | **Absolute difference/mean difference (95% CI)**^a^ | **Risk ratio (95% CI)**^a^ |
| --- | --- | --- | --- | --- | --- | --- | --- |
|  | **N** | **n(%)/mean(SD)** |  | **N** | **n(%)/mean(SD)** |  |  |
| Live birth | 432 | 234 (54.2) |  | 437 | 188 (43.0) | **11.1 (4.5, 17.8)** | **1.26 (1.10, 1.45)** |
| Endometrial thickness (mm) | 423 | 11.0 (1.6) |  | 431 | 10.4 (1.4) | **0.58 (0.39, 0.78)** | ― |
| Cycle cancellation | 432 | 16 (3.7) |  | 437 | 13 (3.0) | 0.7 (-1.7, 3.1) | 1.25 (0.61, 2.56) |
| Biochemical pregnancy | 432 | 294 (68.1) |  | 437 | 260 (59.5) | **8.6 (2.2, 14.9)** | **1.14 (1.03, 1.27)** |
| Clinical pregnancy | 432 | 275 (63.7) |  | 437 | 248 (56.8) | **6.9 (0.4, 13.4)** | **1.12 (1.01, 1.25)** |
| Miscarriage | 275 | 35 (12.7) |  | 248 | 54 (21.8) | **-9 (-15.5, -2.6)** | **0.58 (0.40, 0.86)** |
| Ongoing pregnancy | 432 | 239 (55.3) |  | 437 | 194 (44.4) | **10.9 (4.3, 17.5)** | **1.25 (1.09, 1.43)** |
| Multiple pregnancy | 432 | 18 (4.2) |  | 437 | 19 (4.4) | -0.2 (-2.9, 2.5) | 0.96 (0.51, 1.80) |

NC, natural cycle; HRT, hormone replacement treatment; CI, confidence interval.

^a^ HRT group was regarded as the reference group.
