# Supplementary material for: Natural cycle versus hormone replacement therapy as endometrial preparation in ovulatory women undergoing frozen-thawed embryo transfer: The COMPETE open-label randomized controlled trial
Source: PLoS Med. 2025 Jun 25;22(6):e1004630. doi: 10.1371/journal.pmed.1004630 (PMC12193059; doi:10.1371/journal.pmed.1004630)
Supplement: S5 Table — (DOCX) [file pmed.1004630.s005.docx]

S5 Table. Sensitivity Analysis of Maternal and Perinatal Outcomes by only including First Frozen-thawed Embryo Cycles (Intention-To-Treat)

| **Clinical outcomes** | **NC** | |  | **HRT** | | **Absolute difference/mean difference (95% CI)**^a^ | **Risk ratio (95% CI)**^a^ |
| --- | --- | --- | --- | --- | --- | --- | --- |
|  | **N** | **n(%)/mean(SD)** |  | **N** | **n(%)/mean(SD)** |  |  |
| Maternal hyperthyroidism^*^ | 239 | 6 (2.5) |  | 194 | 3 (1.6) | 1 (-1.7, 3.6) | 1.62 (0.41, 6.41) |
| Maternal hypothyroidism^*^ | 239 | 30 (12.6) |  | 194 | 17 (8.8) | 3.8 (-2, 9.6) | 1.43 (0.81, 2.52) |
| Polyhydramnios^*^ | 233 | 12 (5.2) |  | 188 | 7 (3.7) | 1.4 (-2.5, 5.3) | 1.38 (0.56, 3.44) |
| Oligohydramnios^*^ | 233 | 5 (2.2) |  | 188 | 7 (3.7) | -1.6 (-4.9, 1.7) | 0.58 (0.19, 1.79) |
| Gestational diabetes mellitus | 239 | 26 (10.9) |  | 194 | 31 (16.0) | -5.1 (-11.6, 1.4) | 0.68 (0.42, 1.11) |
| Hypertensive disorders of pregnancy | 239 | 21 (8.8) |  | 194 | 16 (8.3) | 0.5 (-4.7, 5.8) | 1.07 (0.57, 1.98) |
| Pregnancy-induced hypertension | 239 | 13 (5.4) |  | 194 | 12 (6.2) | -0.7 (-5.2, 3.7) | 0.88 (0.41, 1.88) |
| Pre-eclampsia | 239 | 8 (3.4) |  | 194 | 4 (2.1) | 1.3 (-1.7, 4.3) | 1.62 (0.50, 5.31) |
| Antepartum haemorrhage | 234 | 31 (13.3) |  | 188 | 43 (22.9) | **-9.6 (-17, -2.2)** | **0.58 (0.38, 0.88)** |
| Placenta previa | 234 | 2 (0.9) |  | 188 | 2 (1.1) | -0.2 (-2.1, 1.7) | 0.80 (0.11, 5.65) |
| Placenta accreta | 234 | 23 (9.8) |  | 188 | 27 (14.4) | -4.5 (-10.8, 1.8) | 0.68 (0.41, 1.15) |
| Unexplained | 234 | 6 (2.6) |  | 188 | 14 (7.5) | **-4.9 (-9.1, -0.6)** | **0.34 (0.13, 0.88)** |
| Postpartum anemia^*^ | 234 | 16 (6.8) |  | 188 | 16 (8.5) | -1.7 (-6.8, 3.5) | 0.80 (0.41, 1.56) |
| Preterm birth | 239 | 22 (9.2) |  | 194 | 24 (12.4) | -3.2 (-9.1, 2.7) | 0.74 (0.43, 1.29) |
| Spontaneous | 239 | 12 (5.0) |  | 194 | 15 (7.7) | -2.7 (-7.4, 2) | 0.65 (0.31, 1.35) |
| Medical reasons | 239 | 10 (4.2) |  | 194 | 9 (4.6) | -0.5 (-4.4, 3.4) | 0.90 (0.37, 2.18) |
| PPROM^*^ | 233 | 12 (5.2) |  | 188 | 12 (6.4) | -1.2 (-5.7, 3.3) | 0.81 (0.37, 1.75) |
| Mode of delivery, cesarean section^*^ | 234 | 163 (69.7) |  | 188 | 143 (76.1) | -6.4 (-14.9, 2.1) | 0.92 (0.82, 1.03) |
| Gestational age at birth (weeks)^*^ | 234 | 38.8 (1.8)^c^ |  | 188 | 38.7 (1.9) ^b^ | 0.14 (-0.21, 0.49) | ― |
| Singleton |  |  |  |  |  |  |  |
| Birth weight (g) | 224 | 3359.4 (553.9) |  | 173 | 3348.4 (523.1) | 11.00 (-96.60, 118.60) | ― |
| Low birth weight (<2500g) | 224 | 14 (6.3) |  | 173 | 12 (6.9) | -0.7 (-5.6, 4.3) | 0.90 (0.43, 1.90) |
| Very low birth weight (<1500g) | 224 | 1 (0.5) |  | 173 | 1 (0.6) | -0.1 (-1.6, 1.3) | 0.77 (0.05, 12.26) |
| High birth weight (>4000g) | 224 | 19 (8.5) |  | 173 | 13 (7.5) | 1 (-4.4, 6.3) | 1.13 (0.57, 2.22) |
| Very high birth weight (>4500g) | 224 | 3 (1.3) |  | 173 | 1 (0.6) | 0.8 (-1.1, 2.6) | 2.32 (0.24, 22.08) |
| Large for gestational age | 224 | 38 (17.0) |  | 173 | 27 (15.6) | 1.4 (-6, 8.7) | 1.09 (0.69, 1.71) |
| Small for gestational age | 224 | 10 (4.5) |  | 173 | 6 (3.5) | 1 (-2.8, 4.8) | 1.29 (0.48, 3.48) |
| Congenital anomaly | 224 | 7 (3.1) |  | 173 | 5 (2.9) | 0.2 (-3.1, 3.6) | 1.08 (0.35, 3.35) |
| Twins^b^ |  |  |  |  |  |  |  |
| Birth weight (g) | 30 | 2438.7 (382.1) |  | 20 | 2500.5 (395.8) | - 61.83 (-339.21, 215.55) | ― |
| Low birth weight (<2500g) | 30 | 14 (46.7) |  | 20 | 7 (35.0) | 11.7 (-22.8, 46.1) | 0.75 (0.31, 1.80) |
| Very low birth weight (<1500g) | 20 | 0 (0.0) |  | 30 | 0 (0.0) | ― | ― |
| High birth weight (>4000g) | 20 | 0 (0.0) |  | 30 | 0 (0.0) | ― | ― |
| Very high birth weight (>4500g) | 20 | 0 (0.0) |  | 30 | 0 (0.0) | ― | ― |
| Large for gestational age | 20 | 2 (10.0) |  | 30 | 0 (0.0) | ― | ― |
| Small for gestational age | 20 | 5 (25.0) |  | 30 | 2 (6.7) | 18.3 (-4.6, 41.3) | 3.75 (0.78, 17.96) |
| Congenital anomaly | 30 | 4 (13.3) |  | 20 | 4 (20.0) | -6.7 (-37.5, 24.1) | 1.50 (0.24, 9.31) |
| NICU admission^*^ | 233 | 31 (13.3) |  | 189 | 28 (14.8) | -1.5 (-8.2, 5.2) | 0.90 (0.56, 1.44) |
| Perinatal mortality | 0 | 0 (0.0) |  | 0 | 0 (0.0) | ― | ― |

NC, natural cycle; HRT, hormone replacement treatment; CI, confidence interval; PPROM, preterm premature rupture of membranes; NICU, neonatal intensive care unit.

^a^ HRT group was regarded as the reference group.

^b^ Generalized estimating equation for twins. Data represent the number of twin pairs.

^*^ Posthoc specified endpoints.
