# Supplementary material for: Natural cycle versus hormone replacement therapy as endometrial preparation in ovulatory women undergoing frozen-thawed embryo transfer: The COMPETE open-label randomized controlled trial
Source: PLoS Med. 2025 Jun 25;22(6):e1004630. doi: 10.1371/journal.pmed.1004630 (PMC12193059; doi:10.1371/journal.pmed.1004630)
Supplement: S6 Table — (DOCX) [file pmed.1004630.s006.docx]

S6 Table. Sensitivity Analysis of Reproductive Outcomes following cycles with embryo transferred (Intention-To-Treat)

| **Clinical outcomes** | **NC** | |  | **HRT** | | **Absolute difference/mean difference (95% CI)**^a^ | **Risk ratio (95% CI)**^a^ |
| --- | --- | --- | --- | --- | --- | --- | --- |
|  | **N** | **n(%)/mean(SD)** |  | **N** | **n(%)/mean(SD)** |  |  |
| Live birth | 431 | 242 (56.1) |  | 440 | 195 (44.3) | **11.8 (5.2, 18.4)** | 1.27 (1.11, 1.45) |
| Endometrial thickness (mm) | 430 | 10.9 (1.6) |  | 437 | 10.4 (1.3) | **0.57 (0.38, 0.77)** | - |
| Biochemical pregnancy | 431 | 304 (70.5) |  | 440 | 269 (61.1) | **9.4 (3.1, 15.7)** | **1.15 (1.05, 1.27)** |
| Clinical pregnancy | 431 | 285 (66.1) |  | 440 | 257 (58.4) | **7.7 (1.3, 14.1)** | **1.13 (1.02, 1.26)** |
| Miscarriage | 285 | 37 (13.0) |  | 257 | 55 (21.4) | **-8.4 (-14.8, -2.1)** | **0.61 (0.41, 0.89)** |
| Ongoing pregnancy | 431 | 248 (57.5) |  | 440 | 201 (45.7) | **11.9 (5.3, 18.4)** | **1.26 (1.11, 1.43)** |
| Multiple pregnancy | 431 | 18 (4.2) |  | 440 | 19 (4.3) | -0.1 (-2.8, 2.5) | 0.97 (0.51, 1.82) |
| Ectopic pregnancy^*^ | 431 | 6 (1.4) |  | 440 | 7 (1.6) | -0.2 (-1.8, 1.4) | 0.88 (0.30, 2.58) |

NC, natural cycle; HRT, hormone replacement treatment; CI, confidence interval.

^a^ HRT group was regarded as the reference group.

^*^ Posthoc specified endpoints.
