# Supplementary material for: Natural cycle versus hormone replacement therapy as endometrial preparation in ovulatory women undergoing frozen-thawed embryo transfer: The COMPETE open-label randomized controlled trial
Source: PLoS Med. 2025 Jun 25;22(6):e1004630. doi: 10.1371/journal.pmed.1004630 (PMC12193059; doi:10.1371/journal.pmed.1004630)
Supplement: S1 Text — (DOCX) [file pmed.1004630.s007.docx]

# COMPETE TRIAL STATISTICAL ANALYSIS PLAN

Study Title: Comparison of endometrial preparation of natural cycle versus hormone replacement therapy in frozen-thawed embryo transfer cycles (COMPETE): an open-label randomized controlled trial

Short Title: COMPETE Trial

Trial registration: Chinese clinical trial registry number:

ChiCTR2000040640

Funding: The General Projects of Social Development in Shaanxi Province (No. 2022SF-565), and the Xi’an Municipal Science and Technology Bureau, China under grants (24YXYJ0175).

SAP Version v1.0 (final) Date: 10/September/2023

Protocol Version v 3 Date: October 1, 2020

## Introduction

This document is a Statistical Analysis Plan (SAP) for the COMPETE trial. It describes the statistical analyses to be implemented for the analysis of this project. The protocol used as reference for this writing is the current protocol version 3, dated in October 1, 2020. All details of the COMPETE trial can be found in the mentioned protocol. If necessary any future emendations to the protocol, this SAP might be reviewed.

According to the protocol mentioned above, the aim of the study is to compare reproductive, obstetric and perinatal outcomes between natural cycles (NC) and hormone replacement cycles (HRT) in women with a regular ovulatory cycle.

## Study Design and Plan


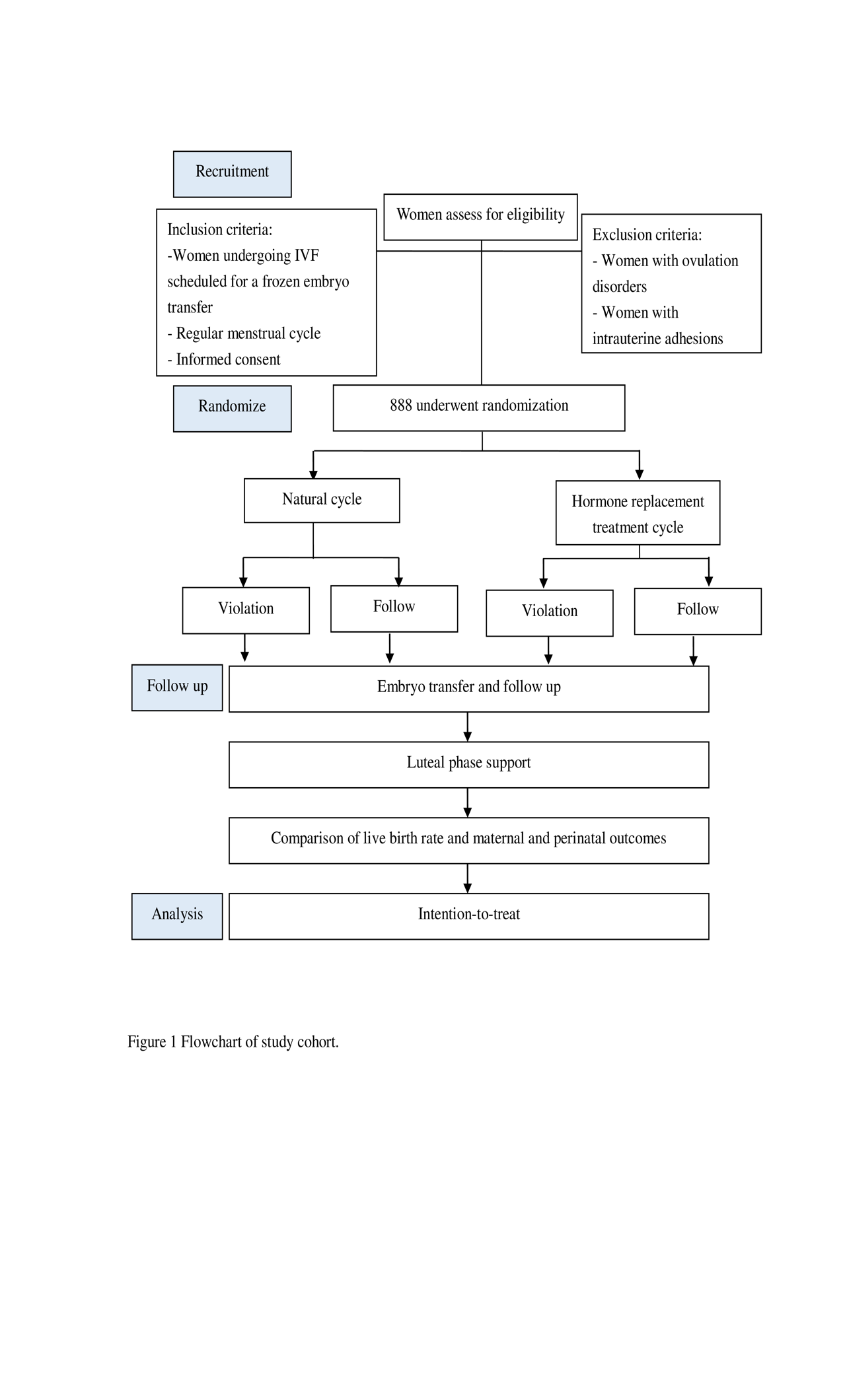


This is a parallel, open-label randomized controlled trial (RCT). For women who are eligible for the study without any exclusion criteria and who accept to participate in the study will be randomized into one of two treatments: natural cycle or hormone replacement cycles, according to the diagram above. Details of inclusion and exclusion criteria are found in sections 4.1 and 4.2.

The primary outcome is live birth resulting from the first FET after randomization after 28 weeks’ gestation. Details will be given in section 5.4.2.

## Sample Size and Power

According to our retrospective cohort study on the same population [6], live birth rates in the HT group was 55.1% in our center. Based on studies within fertility care as well as the discussion by gynaecologist and epidemiologists, we assumed that the minimal clinical important difference to make NC preferable over HT for FET would be 10%. Therefore, we need to include 370 women in each group with two-sided test, 5.0% alpha-error, and 80% statistical power. Assuming a 20% drop out rate, this requires 444 participants in each group. The ratio between intervention and control groups will be 1:1.

.

## Study Population

### 4.1 Inclusion Criteria

The inclusion criteria to participate in the study are women undergoing IVF scheduled for a frozen embryo transfer, having regular menstrual cycle, and giving informed consent.

### 4.2 Exclusion Criteria

Exclusion criteria for the study are women with ovulation disorders or intrauterine adhesions.

## Statistical Analysis Plan (SAP)

### 5.1 General Principles

Data analysis will be performed on the intention-to-treat population and the statistical analysis plan will be finalized prior to data lock. Categorical variables will be expressed by the relative frequency on the corresponding allocation arm. For continuous variables, first quartile, median, and third quartile, or mean and standard deviation will be presented. Missing values will be reported for all variables.

### 5.2 Baseline Characteristics

Baseline characteristics between women will be compared. The number of observations and number of missing values will be reported. Also, baseline variables will be summarized for all patients, and for each one of the treatments applied. Below follows a list of the baseline variables to be considered.

- Female age at freeze
- Female age in study
- Male age
- Infertility duration
- Female BMI
- AFC
- Basal FSH
- Infertility type
- Infertility factors
- Previous pregnancies
- Parity
- Abnormal thyroid stimulating hormone
- Protocol in fresh cycle
- Fertilization type
- Gonadotropin dose
- Gonadotropin duration
- No. of obtained oocytes
- No. of available embryos
- No. of good quality embryos
- Freeze-all policy in fresh cycle
- Live birth in fresh cycle

### 5.3 Analysis

#### 5.3.1 Study populations

Analyses will be performed on the intention to treat principle for all participants. A per protocol principle might be used in some analysis. Here follows predefined protocol violations to define a per protocol population:

- Women assigned to NC treated with HRT or other protocol
- Women assigned to HRT treated with NC or other protocol

The number of women in the intention to treat population and in the per protocol population will be presented. Other protocol violations may be identified during blinded data reviews prior the final analyses.

#### 5.3.2 Efficacy Outcomes

##### 5.3.2.1 Primary Outcomes

The primary outcome is live birth resulting from the first frozen embryo transfer after randomization. Live birth is defined as the delivery of one or more living infants ≥28 week’s gestation.

The response variable is binary indicating whether live birth occurred.

The primary outcome is a binomial random variable. For this outcome, we will estimate risk ratio (RR) with 95% CI. The comparisons of treatments will be performed with a generalized linear model^9^, with the log link function. Risk difference (RD) will also be reported.

To evaluate the potential efficacy of the NC, a per-protocol analysis will also be performed.

##### 5.3.2.2 Secondary Outcomes

To assess the effectiveness of the treatment, we will record these secondary outcomes in terms of effectiveness (from the first transfer after randomization):

- Biochemical pregnancy; defined as serum level of ß-hCG > 50 mIU/ml.
- Clinical pregnancy: defined as one or more observed gestational sac or definitive clinical signs of pregnancy under ultrasonography at 7 weeks after embryo transfer (including clinically documented ectopic pregnancy).
- Multiple pregnancy: defined as a pregnancy with two or more gestational sacs or positive heart beats at 7 weeks of gestation.
- Ongoing pregnancy: defined as the presence of a gestational sac and fetal heartbeat after 12 weeks of gestation.
- Miscarriage (pregnancy loss at <28 weeks).
- Endometrial thickness.
- Cycle cancellation: defined as cancellation of the cycle prior to embryo transfer.

In case of ongoing pregnancy, we will also collect the following obstetric and perinatal complications:

- Gestational diabetes mellitus (GDM)
- Hypertensive disorders of pregnancy (comprising pregnancy induced hypertension (PIH); pre-eclampsia (PET) and eclampsia)
- Antepartum haemorrhage, including placenta previa, placenta accreta and unexplained
- Preterm birth: defined as birth of a fetus delivered after 28 and before 37 completed weeks of gestational age in participants confirmed ongoing pregnancy. We will also collect causes of preterm birth; i.e. spontaneous and iatrogenic delivery.
- Birth weight, including low birth weight (defined as weight < 2500 gm at birth), very low birth weight (defined as < 1500 gm at birth), high birth weight (defined as >4000 gm at birth) and very high birth weight (defined as >4500 gm at birth)
- Large for gestational age (defined as birth weight >90th centile for gestation, based on standardized ethnicity-based charts) and small for gestational age (defined as less than 10th centile for gestational age at delivery based on standardized ethnicity-based charts); birthweight percentage.
- Congenital anomaly (any congenital anomaly will be included)
- Perinatal mortality: defined as fetal or neonatal death occurring during late pregnancy (at 24 completed weeks of gestational age and later), during childbirth, or up to seven completed days after birth.

Secondary outcomes will be compared between the two arms using the similar approach described for the primary outcome. However, the outcomes of twins will be analyzed using the general estimating equation to factor in the correlation between twins.

##### 5.3.2.3 Post hoc Outcomes

To further assess the effectiveness and safety of the treatment, we will report on the following post hoc outcomes that were not described in the protocol.

- Ectopic pregnancy: defined as a pregnancy that occurs outside of the uterine cavity.
- Mode of delivery: included cesarean section and vaginal delivery.
- Gestational age at birth: defined as duration of pregnancy before birth.
- Postpartum anemia: hemoglobin of <110 g/L at 1 week postpartum.
- Maternal hyperthyroidism: TSH below the lower reference limit (i.e. the 2.5^th^ percentile) and FT4 above the upper reference limit (i.e. the 97.5^th^ percentile) from the guidelines on diagnosis and management of thyroid diseases during pregnancy for Chinese pregnant women [9].
- Maternal hypothyroidism: TSH above the upper reference limit and FT4 below the lower reference limit.
- Polyhydramnios: defined as an amniotic fluid index>95^th^ percentile or a deepest vertical pocket≥8 cm.
- Oligohydramnios: defined as an amniotic fluid index<5^th^ percentile or a deepest vertical pocket≤5 cm.
- Neonatal care intensive unit (NICU) admission.

### 5.4 Complementary analyses

#### 5.4.1 Subgroups

The primary outcome will be compared between the two arms within several clinically important subgroups including female age groups (<35 / >=35 years), freeze-all policy in fresh cycles, and embryo stage in which the effects on outcomes might be modified. Due to the concern over multiplicity of sub-group analysis, we will place limited importance on subgroup findings.

#### 5.4.2 Sensitivity analysis

The researchers are aware that a small number of second frozen embryo transfer cycles (around 3% of total sample size) were randomized because their first frozen embryo transfers were unsuccessful and erroneously reentered into the screening pool. This may have minimal impact on the independence between observations because the proportion is small. However, we will perform a sensitivity analysis that only includes first frozen transfer cycles.

#### 5.4.3 Safety Outcomes

##### 5.4.3.1 Treatment Compliance

Patients are expected to return the outpatient department at each visit. Patients should follow the protocol from the date of randomization until the end of the treatment.

Compliance will be calculated as the ratio of the number of women received the allocated protocol divided by the number of women allocated to this protocol. Compliance will be summarized for all women and separately for both treatment groups.

##### 5.4.3.2 Premature Withdrawal

The following details on premature withdrawals will be summarized according to treatment groups:

- Number of women who stopped treatment
- Main reason for discontinuation

- Woman unwilling to continue

- Severe adverse event

- Woman violated protocol

- Investigator terminated participation

- Woman withdrawn consent for use of outcome data

- Other reason

### 5.5 Additional Analyses

Additional analyses of those specified in this SAP based on the results of the primary and secondary analyses may be carried out at a later when appropriate.

## References

1. Shi, Y., et al., *Transfer of Fresh versus Frozen Embryos in Ovulatory Women.* N Engl J Med, 2018. **378**(2): p. 126-136.

2. Fazleabas, A.T. and Z. Strakova, *Endometrial function: cell specific changes in the uterine environment.* Mol Cell Endocrinol, 2002. **186**(2): p. 143-7.

3. Groenewoud, E.R., et al., *What is the optimal means of preparing the endometrium in frozen-thawed embryo transfer cycles? A systematic review and meta-analysis.* Hum Reprod Update, 2017. **23**(2): p. 255-261.

4. Glujovsky, D., et al., *Endometrial preparation for women undergoing embryo transfer with frozen embryos or embryos derived from donor oocytes.* Cochrane Database Syst Rev, 2020. **10**(10): p. Cd006359.

5. Singh, B., et al., *Frozen-thawed embryo transfer: the potential importance of the corpus luteum in preventing obstetrical complications.* Fertil Steril, 2020. **113**(2): p. 252-257.

6. Liu, X., W. Shi, and J. Shi, *Natural cycle frozen-thawed embryo transfer in young women with regular menstrual cycles increases the live-birth rates compared with hormone replacement treatment: a retrospective cohort study.* Fertil Steril, 2020. **113**(4): p. 811-817.

7. Mounce, G., et al., *Randomized, controlled pilot trial of natural versus hormone replacement therapy cycles in frozen embryo replacement in vitro fertilization.* Fertil Steril, 2015. **104**(4): p. 915-920.e1.

8. Madani, T., et al., *Live birth rates after different endometrial preparation methods in frozen cleavage-stage embryo transfer cycles: a randomized controlled trial.* 2019. **299**(4): p. 1185-1191.

9. AHW, C., *Guideline on diagnosis and management of thyroid diseases during pregnancy and postpartum (2nd edition).* Chin J Periat Med, 2019(8): p. 636-665.
