# Supplementary material for: Natural cycle versus hormone replacement therapy as endometrial preparation in ovulatory women undergoing frozen-thawed embryo transfer: The COMPETE open-label randomized controlled trial
Source: PLoS Med. 2025 Jun 25;22(6):e1004630. doi: 10.1371/journal.pmed.1004630 (PMC12193059; doi:10.1371/journal.pmed.1004630)
Supplement: S3 Text — (DOCX) [file pmed.1004630.s009.docx]

**Supplement**

**Hormone replacement therapy versus natural cycle as endometrial preparation in women undergoing frozen-thawed embryo transfer: the COMPETE randomized controlled trial**

# COMPETE TRIAL PROTOCOL

**VERSION 3**

**(Nov. 2020)**

**Natural Cycle and Hormone Replacement Cycle Endometrial Preparation Protocols for Frozen Embryo Transfer**

**A randomized controlled trial**

| Protocol ID |  |
| --- | --- |
| Short title | Comparison of different Endometrial Preparation Protocols for Frozen Embryo Transfer: a randomized controlled trial |
| Version | 3.0 |
| Date | Nov 2020 |
| Coordinating investigator/project leader | Dr. Xitong Liu  Northwest Women's and Children's Hospital  Xi’an, China |
| Principal investigators | Dr. Juanzi Shi  Northwest Women's and Children's Hospital  Xi’an, China  Prof. B.W.J. Mol  Department of Obstetrics and Gynaecology  Monash Medical Centre  Monash University  Melbourne, Australia  Dr. Wentao Li  Department of Obstetrics and Gynaecology  Monash Medical Centre  Monash University  Melbourne, Australia |
| Collaborative investigators | Dr Wen Wen  Northwest Women's and Children's Hospital  Xi’an, China  Dr Tao Wang  Northwest Women's and Children's Hospital  Xi’an, China  Dr Ting Sun  Northwest Women's and Children's Hospital  Xi’an, China  Dr Ting Wang  Northwest Women's and Children's Hospital  Xi’an, China  Dr Na Zhang  Northwest Women's and Children's Hospital  Xi’an, China  Dr Dan Pan  Northwest Women's and Children's Hospital  Xi’an, China  Dr He Cai  Northwest Women's and Children's Hospital  Xi’an, China  Dr Jinlin Xie  Northwest Women's and Children's Hospital  Xi’an, China  Dr Xiaojuan Liu  Northwest Women's and Children's Hospital  Xi’an, China  Dr Zan Shi  Northwest Women's and Children's Hospital  Xi’an, China  Dr Rui Wang  Northwest Women's and Children's Hospital  Xi’an, China  Dr Xiaofang Li  Northwest Women's and Children's Hospital  Xi’an, China  Dr Na Lu  Northwest Women's and Children's Hospital  Xi’an, China  Dr Rong Pan  Northwest Women's and Children's Hospital  Xi’an, China  Dr Li Tian  Northwest Women's and Children's Hospital  Xi’an, China  Dr Bin Meng  Northwest Women's and Children's Hospital  Xi’an, China  Dr Haiyan Bai  Northwest Women's and Children's Hospital  Xi’an, China  Dr Hanying Zhou  Northwest Women's and Children's Hospital  Xi’an, China  Pro. Pengfei Qu  Northwest Women's and Children's Hospital  Xi’an, China  Pro. Doudou Zhao  Northwest Women's and Children's Hospital  Xi’an, China  Pro. Danmeng Liu  Northwest Women's and Children's Hospital  Xi’an, China |
| Sponsor | This trial was funded by the General Projects of Social Development in Shaanxi Province, without any commercial support. |
| Collaborate Institutes | None |
|  |  |

**Content**

[1. INTRODUCTION AND RATIONALE 8](#_Toc87866637)

[2. OBJECTIVE 9](#_Toc87866638)

[3. STUDY DESIGN 9](#_Toc87866639)

[4. STUDY POPULATION 10](#_Toc87866640)

[4.1 Inclusion and exclusion criteria 10](#_Toc87866641)

[4.2 Sample size calculation 11](#_Toc87866642)

[5. RECRUITMENT, RANDOMIZAITON, INTERVENTIONS, AND PROCEDURES 11](#_Toc87866643)

[5.1 Recruitment 11](#_Toc87866644)

[5.3 Interventions 12](#_Toc87866645)

[6. METHODS 13](#_Toc87866646)

[6.1 Study endpoints 13](#_Toc87866647)

[6.2 Subject informed consent 15](#_Toc87866648)

[6.3 Withdrawal of individual participants 15](#_Toc87866649)

[6.4 Duration of the study 15](#_Toc87866650)

[6.5 Statistical analysis 15](#_Toc87866651)

[6.6 Data and Safety Monitoring Board 17](#_Toc87866652)

[6.7 Interim analysis 17](#_Toc87866653)

[7. SAFETY REPORTING 17](#_Toc87866654)

[7.1 WMO event 17](#_Toc87866655)

[7.2 Adverse and serious adverse events 18](#_Toc87866656)

[7.3 Follow-up of adverse events 18](#_Toc87866657)

[8. ETHICAL CONSIDERATIONS 18](#_Toc87866658)

[8.1 Regulation statement 18](#_Toc87866659)

[8.2 Recruitment and consent 19](#_Toc87866660)

[8.3 Privacy 19](#_Toc87866661)

[8.4 Benefits and risks assessment, group relatedness 19](#_Toc87866662)

[9. FEASIBILITY OF STUDY 20](#_Toc87866663)

[10. ADMINISTRATIVE ASPECTS AND PUBLICATION 20](#_Toc87866664)

[10.1 Handling and storage of data and documents 20](#_Toc87866665)

[10.2 Annual progress report 20](#_Toc87866666)

[10.3 End of study report 21](#_Toc87866667)

[10.4 Public disclosure and publication policy 21](#_Toc87866668)

[11 REFERENCES 21](#_Toc87866669)

# 1. INTRODUCTION AND RATIONALE

With the development of in vitro fertilization-embryo transfer (IVF-ET) technology, frozen-thawed embryo transfer (FET) has been widely used because it can provide a more physiologic uterine environment and prevent ovarian hyperstimulation syndrome (OHSS), facilitate single embryo transfer and improve fertility and pregnancy outcomes [1]. The synchronization of embryo and endometrium development plays an important role in implantation [2]. Endometrial preparation is of utmost importance for frozen embryo transfer to optimize pregnancy rates. At present, the commonly used FET endometrial preparation protocols include the natural cycle (NC) and the hormone replacement cycle (HT). A natural cycle involves a dominant follicle matures and produces estradiol which leads to endometrium thickening. In contrast, exogenous estradiol and progesterone were administered to prepare endometrium development in a hormone replacement cycle.

While several retrospective studies have investigated the clinical outcomes between different methods of endometrial preparation, it is still unclear what is optimal means of preparing the endometrium in frozen–thawed embryo transfer cycles [3, 4]. In theory, the hormone replacement cycle requires medication, and it might be less physiological than a natural ovulatory cycle. In addition, recent studies suggested that the absence of the corpus luteum (CL) in hormone replacement cycles were responsible for the increased risk of maternal complications like hypertensive disorders of pregnancy [5].

We performed a retrospective cohort study comparing natural cycle and hormone replacement treatment cycle in women < 35 years old with regular menstrual cycles, and found natural cycle has a higher live birth and lower miscarriage rate than hormone replacement treatment cycle [6]. Currently, there are few randomized controlled studies on the application of these two protocols in women with regular menstrual cycles. One open, single-center, randomized controlled trial compared natural and down-regulated hormone replacement treatment, and found women with ovulatory cycles had similar live birth rate between two groups [7]. However, this RCT only included 159 patients, lacking the power to draw firm conclusions. Another RCT randomized women into four groups of endometrial preparation: natural cycle with or without human chorionic gonadotropin (hCG), hormone replacement treatment with or without pre-treatment with gonadotropin releasing hormone agonist (GnRHa), and no significant difference were found in terms of pregnancy, miscarriage and live birth between four groups [8]. However, this study was also underpowered and suffer from methodological weaknesses with incomplete reporting of the study design and type of randomization.

Therefore, we have developed this large-sample randomized controlled trial to compare these two protocols. We hypothesise that natural cycle leads to a higher live birth rate than the hormone replacement treatment cycle.

# 2. OBJECTIVE

We propose a randomized controlled clinical trial, to compare the effectiveness of a NC versus HT endometrial preparation protocol on live birth rates in women with regular menstrual cycles who undergoing the first FET cycles.

# 3. STUDY DESIGN

A single centre, parallel, open-label, randomized controlled clinical trial (1:1 treatment ratio). The flowchart of the study is shown in Figure 1.

Follow

Violation

Embryo transfer and follow up

Women assess for eligibility

Recruitment

Inclusion criteria:

-Women undergoing IVF scheduled for a frozen embryo transfer

- Regular menstrual cycle

- Informed consent

Exclusion criteria:

- Women with ovulation disorders

- Women with intrauterine adhesions

888 underwent randomization

Hormone replacement treatment cycle

Natural cycle

Follow

Violation

Randomize

Luteal phase support

Comparison of live birth rate and maternal and perinatal outcomes

Intention-to-treat

Follow up

Analysis

Figure 1 Flowchart of study cohort.

# 4. STUDY POPULATION

## 4.1 Inclusion and exclusion criteria

Inclusion Criteria:

- Women undergoing IVF scheduled for a frozen embryo transfer
- Regular menstrual cycle
- Informed consent

Exclusion criteria:

- Women with ovulation disorders.
- Women with intrauterine adhesions

## 4.2 Sample size calculation

According to our retrospective cohort study [1], live birth rates was 55.1% in the hormone replacement cycle. Based on studies within fertility care as well as the discussion by gynaecologist and epidemiologists, we assumed that the minimal clinical important difference to make natural cycle preferable over hormone replacement cycle of FET would be 10%. To demonstrate a 10% difference, we need to include 370 women in each group with two-sided test, 5.0% alpha-error, 80% statistical power. Assuming a 20% drop out rate this requires 444 participants in each group. The ratio between intervention and control groups will be 1:1.

After recruiting 444 participants, an interim analysis will be performed. Criteria will be specified in the statistical analysis plan.

# 5. RECRUITMENT, RANDOMIZATION, INTERVENTIONS, AND PROCEDURES

## 5.1 Recruitment

Infertile women undergoing IVF-ET who come to the outpatient clinic to receive FET will be screened by trained clinical team. Eligible women will then be explained the trial details by a member of the research team and before the start of FET treatment. If a woman agrees to participate, she will sign the consent form. An individual record of all non-recruited women and reasons for exclusion will be obtained and stored.

***5.1 Randomization***

Patients fulfilling eligibility criteria and willing to participate will be allocated randomly into one of the two arms at a ratio of 1:1 on menstrual cycle day 5. Computer-generated random numbers will be used to allocate patients into two groups. Simple randomization will be centrally controlled by using web-based electronic data capture (ResMan). Both the investigators and patients will be aware of the allocation. The embryologists and doctors involved in the embryo transfer are blinded to the group assignments of the patients in the trial.

## 5.3 Interventions

## Group A: Natural cycle

Women in the natural cycle group will undergo transvaginal ultrasound from day 5 of the menstrual cycle. Follicular growth will be monitored through transvaginal ultrasound and serum luteinizing hormone (LH). When LH>20 IU/L, transvaginal ultrasound will be performed daily until ovulation. If the leading follicle reaches a mean diameter of >17mm while LH <20 IU/L, 10000 IU of human chorionic gonadotropin (hCG) will be administered to trigger oocytes ovulation. When the ovulation is confirmed by transvaginal ultrasound, 200 mg of vaginal micronized progesterone will be initiated three times a day and continued for 3 days for cleavage stage embryos and 5 days for blastocyst stage embryos.

## Group B:HT cycle

Endometrial preparation will be initiated with oral estradiol valerate (Progynova; Bayer Schering Pharma AG, Berlin, Germany) at a daily dose of 6mg from day 5 of menstrual cycle. A transvaginal ultrasound and serum progesterone level will be performed 10-12 days later. Provided the endometrial thickness reached 7mm or more and P <1.5 ng/ml, 200 mg of vaginal micronized progesterone will be initiated three times a day and continued for 4 days for cleavage stage embryos and 5 days for blastocyst stage embryos.

*Embryo transfer and luteal support*

To reduce the risk of high-order multiple pregnancies, the number of embryos transferred will be mostly limited to one or two best-quality embryos. Luteal support is administered in the form of vaginal progesterone (90mg q.d.; Crinone, Serono, Hertfordshire, UK) or vaginal progesterone soft capsules (0.2g t.i.d; Utrogestan, Besins, France) or intramuscular progesterone (60mg q.d.; Zhejiang, Xianju, China), oral progesterone (10mg t.i.d.; Dydrogesterone, Abbott Biologicals B.V., Netherlands) , and estradiol vaterate (3mg b.i.d.; Progynova, Bayer, France) until the confirmation of biochemical pregnancy, and will be maintained to 10 weeks of gestation. The progesterone will be used until the menses when the biochemical pregnancy is not observed.

Women who cancel the embryo transfer cycle will follow local protocol for the next cycle. They will still be accounted for in the intention-to-treat analysis.

# 6. METHODS

## 6.1 Study endpoints

Primary outcome

Our primary outcome will be live birth resulting from the first frozen embryo transfer after randomization. Live birth is defined as a delivery of one or more living infants ≥28 week’s gestation.

Secondary outcomes

To assess the effectiveness of the treatment, we will record these secondary outcomes in terms of effectiveness (from the first transfer after randomization):

- Biochemical pregnancy; defined as serum level of ß-hCG > 50 mIU/ml.
- Clinical pregnancy: defined as one or more observed gestational sac or definitive clinical signs of pregnancy under ultrasonography at 7 weeks after embryo transfer (including clinically documented ectopic pregnancy).
- Multiple pregnancy: defined as a pregnancy with two or more gestational sacs or positive heart beats at 7 weeks of gestation.
- Ongoing pregnancy: defined as the presence of a gestational sac and fetal heartbeat after 12 weeks of gestation.
- Miscarriage (pregnancy loss at <28 weeks).
- Endometrial thickness.
- Cycle cancellation: defined as cancellation of the cycle prior to embryo transfer.

In case of ongoing pregnancy, we will also collect the following obstetric and perinatal complications:

- Gestational diabetes mellitus (GDM)
- Hypertensive disorders of pregnancy (comprising pregnancy induced hypertension (PIH); pre-eclampsia (PET) and eclampsia)
- Antepartum haemorrhage, including placenta previa, placenta accreta and unexplained
- Preterm birth: defined as birth of a fetus delivered after 28 and before 37 completed weeks of gestational age in participants confirmed ongoing pregnancy. We will also collect causes of preterm birth; i.e. spontaneous and iatrogenic delivery.
- Birth weight, including low birth weight (defined as weight < 2500 gm at birth), very low birth weight (defined as < 1500 gm at birth), high birth weight (defined as >4000 gm at birth) and very high birth weight (defined as >4500 gm at birth)
- Large for gestational age (defined as birth weight >90th centile for gestation, based on standardized ethnicity-based charts) and small for gestational age (defined as less than 10th centile for gestational age at delivery based on standardized ethnicity-based charts); birthweight percentage.
- Congenital anomaly (any congenital anomaly will be included)
- Perinatal mortality: defined as fetal or neonatal death occurring during late pregnancy (at 24 completed weeks of gestational age and later), during childbirth, or up to seven completed days after birth.

## 6.2 Subject informed consent

Infertile couples scheduled for IVF who come to the outpatient clinic to receive FET will be screened by trained clinical team. Eligible women will then be approached by a member of the research team and explained the trial details before the start of FET treatment. After this information, couples will be offered time for consideration to decide to participate the trial. If the couple agrees to participate, they will sign the consent form. An individual record of all non-recruited participants and reasons for exclusion will be obtained and stored.

## 6.3 Withdrawal of individual participants

Participants have the right to withdraw from at any time during the process. The decision to withdraw will neither affect their conventional clinical treatments nor their relationship with clinicians.

## 6.4 Duration of the study

The recruitment in the study centre will start in Dec 2020 and will continue until the needed number of participants is included, anticipated until Dec 2022. The study duration is therefore estimated to be two years.

## 6.5 Statistical analysis

A separate statistical analysis plan will be approved before the last participant is included. The statistical analysis will be performed by an independent statistician, overseen by investigators at Monash University.

*Baseline data*

Baseline characteristics will be described by descriptive analysis, and the balance between the two arms will be assessed. For continues variables, the normality test will be estimated using frequency histograms and the Shapiro test initially. If the parameters are non-normally distributed, their medians and inter-quantile ranges (IQRs) will be reported. For categorical variables, we will present the proportions of the two arms. In addition, we will also report the numbers of recruitment, participants lost to follow-up, protocols violation, and other relevant descriptive data.

*Primary outcome*

Data analysis of this trial will be analysed according to the intention-to-treat principle. We will include all randomized women in the primary comparison between the two arms. Per-protocol analysis may be conducted as a secondary analysis. The primary outcome, live birth rate, will be compared between the two arms using Pearson’s chi-square test or Fisher’s exact test for unadjusted analysis. We will also compute unadjusted risk ratio (RR) and its 95% confidence interval (95% CI). In the event of prominent imbalance of potential confounders between the two arms, these characteristics will be adjusted in multivariable models.

*Secondary outcomes*

Secondary outcomes will be compared between the two arms using the similar approach described for the primary outcome.

*Subgroup analysis*

Primary and secondary outcomes will be compared between the two arms within several clinically important subgroups including female age groups (<35 / >=35 years), freeze-all policy in fresh cycles, and embryo stages (cleavage / blastocyst) in which the effects on outcomes might be modified. Due to the concern over multiplicity of sub-group analysis, we will place limited importance on subgroup findings.

*Missing data and sensitivity analysis*

For missing values regarding baseline characteristics, we will first perform analysis by excluding missing values, we will then perform multiple imputation to impute missing values and conduct subsequent analysis to estimate the robustness of the findings. For loss to follow-up and protocol violation, we will attempt sensitive analyses to explore the effect of these factors on the trial findings.

*Interim analysis plan*

The ongoing pregnancy and miscarriage will be calculated as effectiveness of the study with risk difference and risk ratio. Each number of women completed the follow up will be recorded. AE and SAE will be recorded.

All tests will be two-tailed, and differences with p value <0.05 will be considered statistically significant if the Bonferroni correction is not applied.

## 6.6 Data and Safety Monitoring Board

We will establish an independent Data and Safety Monitoring Board (DSMB) to review and interpret data generated from the study and to review revisions of the protocol prior to their implementation. Its primary objectives are to ensure the safety of study subjects and the integrity of the research data. The DSMB advises on research design issues, data quality and analysis, and research participant protections for the study.

| Yuhua Shi | Chair of DSMB |
| --- | --- |
| Cuifang Hao | Member of DSMB |
| Yihong Guo | Member of DSMB |

The DSMB will hold regular conference calls to review the protocol with respect to ethical and safety standards, monitor the safety of the trials, monitor the integrity of the data with respect to original study design, and provide advice on study conduct. The DSMB will review the progress of the trial, adjudicate adverse events, and decide on any premature closure of the study. The DSMB consists of three members. Voting members consist of individuals who are impartial, independent of the investigator(s), and who have no financial, scientific, or other conflict of interests with the study.

## 6.7 Interim analysis

An interim analysis is planned to be organized within four months after recruiting 444 participants. The DSMB will be asked to assess the endpoint on ongoing pregnancy, as data on live birth will not be available at the interim analysis. Also, the DSMB will be provided insight in the SAE’s that have occurred. The study could be stopped prematurely based on the advice of the DSMB.

# 7. SAFETY REPORTING

## 7.1 WMO event

The investigator will inform subjects and the reviewing accredited medical research ethics committee if anything occurs, on the basis of which it appears that the disadvantages of participation may be significantly greater than was foreseen in the research proposal. The study will be suspended pending further review by the accredited medical research ethics committee, unless suspension would jeopardize the subjects’ health. The investigator will take care that all subjects are kept informed.

## 7.2 Adverse and serious adverse events

All observed or volunteered adverse events, regardless of treatment group or suspected causal relationship to intervention, will be recorded. Adverse events are defined as any undesirable experience occurring to a subject during the trial, whether or not considered related to the intervention. All adverse events reported spontaneously by the subject or observed by the investigator or their staff will be recorded.

A serious adverse event (SAE) is any untoward medical occurrence or effect, at any dose, that results in death; is life threatening (at the time of the event); requires hospitalization or prolongation of existing inpatients’ hospitalization; results in persistent or significant disability or incapacity; is a congenital anomaly or birth defect; is a new event of the trial likely to affect the safety of the subjects, such as an unexpected outcome of an adverse reaction. All SAEs will be reported to the DSMB and accredited Medical Education Technology Committee (METC) that approved the protocol, according to the requirements of that METC.

## 7.3 Follow-up of adverse events

All adverse events will be followed until they have abated, or until a stable situation has been reached. Depending on the event, follow-up may require additional tests or medical procedures as indicated, and/or referral to a general physician or a medical specialist.

# 8. ETHICAL CONSIDERATIONS

## 8.1 Regulation statement

The study will be conducted according to the principles of the Declaration of Helsinki (World Medical Association Declaration Of Helsinki Ethical Principles for Medical Research Involving Human Subjects Version Edinburgh, Scotland, October 2000, with Note of Clarification on Paragraph 29 added by the WMA General Assembly, Washington 2002 end Note of Clarification on Paragraph 30 added by the WMA General Assembly, Tokyo 2004) and in accordance with the Medical Research Involving Human Subjects Act (WMO) and other guidelines, regulations and Acts.

## 8.2 Recruitment and consent

The investigator must explain to each subject the nature of this study, its purpose, procedures, expected duration and the potential risks and benefits involved in study participation along with any discomfort it may entail. Each subject must be informed that participation in the study is voluntary and that withdrawal of consent will not affect her right to the most appropriate medical treatment or affect the doctor relationship.

Informed consent will be given by means of a standard written statement. It will be written so as to be easily understood by the subject. The subject will be given the time to read and understand the statement herself before signing her consent and dating the document. The subject will be provided with a copy of the written statement once signed.

## 8.3 Privacy

Each participant will be assigned an appropriate code number that is consistent with the allocated intervention, which will appear on all report forms to maintain confidentiality.

## 8.4 Benefits and risks assessment, group relatedness

Participants who are entered into the ovarian response screening and possible subsequent randomization will undergo extra diagnostic testing. This will include one extra hospital visit to undergo these investigations. The benefit for the participants in the experimental arms could be a better balance between success rates and women’s discomfort in the sequence

of IVF/ICSI treatment.

# 9. FEASIBILITY OF STUDY

In the study center, approximately 15000 cycles are performed per year. The number of couples who undergo IVF/ICSI cycle is approximately 9000 per year. With the numerous numbers of participants and cycles, adequate participant enrolment will be ensured.

# 10. ADMINISTRATIVE ASPECTS AND PUBLICATION

## 10.1 Handling and storage of data and documents

The data collected for the trial will be a mixture of routinely clinical data (such as demographic data, fertility history, ART records), which are verifiable from the medical record and questionnaire data. To guarantee the authentic study results, all of the researchers and physicians in our study are required to receive training classes and pass the test. They must master all details of this trial before preforming it, as well as the randomization method and case report form (CRF).

All the characteristic mentioned above are collected at baseline and follow-up through a standard clinical electronic data collection system (EDC). Initially, each study site will be required to keep accurate and verifiable source notes in the medical record relevant to each participant’s eligible criteria of this trial. After recruitment of eligible participants, trained assessors will take charge of the data input: they can log on to a secure data portal with the individual ID for each site, and upload the data from medical record to eCRF with the personal trail ID of each participant. When the trial is close-out, all participant-identifiable data, such as consent forms, screening and identification logs will be stored in the investigator site files, accessible only to delegated members of the study team.

## 10.2 Annual progress report

The principal investigator will submit a summary of the progress of the trial to the accredited METC once a year. Information will be provided on the date of inclusion of the first subject, numbers of subjects included and numbers of subjects that have completed the trial, SAEs/serious adverse reactions, other problems, and amendments.

## 10.3 End of study report

The principal investigator will notify the accredited METC and the competent authority of the end of the study within a period of 90 days. The end of the study is defined as the last participant’s last visit. In case the study is ended prematurely, the principal investigator will notify the accredited METC within 15 days, including the reasons for the premature termination.

## 10.4 Public disclosure and publication policy

The researchers will permit trial-related monitoring, audits, regulatory inspections, providing direct access to source data and documents. The principal investigator will publish the results of the study as soon as appropriate*.*

**11 REFERENCES**

1. Shi, Y., et al., *Transfer of Fresh versus Frozen Embryos in Ovulatory Women.* N Engl J Med, 2018. **378**(2): p. 126-136.

2. Fazleabas, A.T. and Z. Strakova, *Endometrial function: cell specific changes in the uterine environment.* Mol Cell Endocrinol, 2002. **186**(2): p. 143-7.

3. Groenewoud, E.R., et al., *What is the optimal means of preparing the endometrium in frozen-thawed embryo transfer cycles? A systematic review and meta-analysis.* Hum Reprod Update, 2017. **23**(2): p. 255-261.

4. Glujovsky, D., et al., *Endometrial preparation for women undergoing embryo transfer with frozen embryos or embryos derived from donor oocytes.* Cochrane Database Syst Rev, 2020. **10**(10): p. Cd006359.

5. Singh, B., et al., *Frozen-thawed embryo transfer: the potential importance of the corpus luteum in preventing obstetrical complications.* Fertil Steril, 2020. **113**(2): p. 252-257.

6. Liu, X., W. Shi, and J. Shi, *Natural cycle frozen-thawed embryo transfer in young women with regular menstrual cycles increases the live-birth rates compared with hormone replacement treatment: a retrospective cohort study.* Fertil Steril, 2020. **113**(4): p. 811-817.

7. Mounce, G., et al., *Randomized, controlled pilot trial of natural versus hormone replacement therapy cycles in frozen embryo replacement in vitro fertilization.* Fertil Steril, 2015. **104**(4): p. 915-920.e1.

8. Madani, T., et al., *Live birth rates after different endometrial preparation methods in frozen cleavage-stage embryo transfer cycles: a randomized controlled trial.* 2019. **299**(4): p. 1185-1191.
