# Supplementary material for: Natural cycle versus hormone replacement therapy as endometrial preparation in ovulatory women undergoing frozen-thawed embryo transfer: The COMPETE open-label randomized controlled trial
Source: PLoS Med. 2025 Jun 25;22(6):e1004630. doi: 10.1371/journal.pmed.1004630 (PMC12193059; doi:10.1371/journal.pmed.1004630)
Supplement: Attachment — Submitted filename: PLOS review.docx [file pmed.1004630.s011.docx]

In this open label RCT, in women with a regular menstrual cycle undergoing IVF, the live birth rate following frozen embryo transfer (FET) in and natural cycle was compared with the live birth rate following FET in a programmed HRT cycle. The intention to treat analysis indicated that natural cycle preparation for FET was associated with 54% live birth rate whereas transfer following HRT preparation resulted in a significantly lower chance of achieving a live birth rate at 43% in the HRT group.

The rationale for performing such a trial is understandable given most literature on the subject have not addressed the question whether a particular method of preparation for FET is superior to the other. However, the findings of this raise at least two pertinent questions a question. The first one is about biological plausibility of achieving this remarkably high live birth rate following FET whether it is following natural cycle or after HRT preparation. The live births rates reported here following frozen FET have not be reported by any previous study on the subject to date. Moreover, the significant difference between the natural and HRT prepared FET has not previously been reported in any other RCT. In fact, a recently published, adequately powered study, no significant difference in the livebirth outcome between natural and HRT prepared FET was found. The livebirth rate after one FET was 174 (37%) of 476 in the natural cycle strategy group, 159 (33%) of 476 in the modified natural cycle strategy group, and 162 (34%) of 476 in the artificial cycle strategy group (1).

It is interesting such high live birth rate and the significant difference in outcome of FET in favour of natural cycle FET were only in addition to this manuscript, was reported in published cohort and retrospective studies from the centre where this current study has been conducted (2)

The above observations question the biological plausibility of the result and raises a question regarding potential intellectual bias, both of which make a case for examining the raw data which I hereby renew my request to make available in order to complete an objective assessment of the data underpinning the findings of this study.

*1-Vu N A Ho, et.al.,* ***Lancet* 2024; 404: 266–75**

2-liu X, Sh W, SheJ Fertil Steril. 2020:113 (4):811-7
